# Experiential peer support and its effects on desistance from delinquent behavior: protocol paper for a systematic realist literature review

**DOI:** 10.1186/s13643-019-1036-2

**Published:** 2019-05-18

**Authors:** Margriet Lenkens, Frank J. van Lenthe, Loïs Schenk, Tessa Magnée, Miranda Sentse, Sabine Severiens, Godfried Engbersen, Gera E. Nagelhout

**Affiliations:** 1 IVO Research Institute, P.O. Box 30833, 2500 GV The Hague, The Netherlands; 2 Department of Public Health, Erasmus Medical Center, P.O. Box 2040, 3000 CA Rotterdam, The Netherlands; 3 Department of Psychology, Education and Child Studies, Erasmus School of Social and Behavioural Sciences, Erasmus University Rotterdam, P.O. Box 1738, 3000 DR Rotterdam, The Netherlands; 4 Department of Criminology, Faculty of Law, Leiden University, P.O. Box 9520, 2300 RA Leiden, The Netherlands; 5 Department of Public Administration and Sociology, Erasmus School of Social and Behavioural Sciences, Erasmus University Rotterdam, P.O. Box 1738, 3000 DR Rotterdam, The Netherlands; 6 Department of Health Promotion and Department of Family Medicine, Maastricht University (CAPHRI), P.O. Box 616, 6200 MD Maastricht, The Netherlands; 7 FortaGroep, Kruisplein 25F, 3014 DB Rotterdam, The Netherlands

**Keywords:** Protocol, Realist review, Delinquent behavior, Criminal behavior, Peer support, Experiential peer, Desistance, Rehabilitation, Experiential expertise

## Abstract

**Background:**

Experiential peers are increasingly involved in the development and delivery of interventions for individuals who are engaged in delinquent behavior. Experiential peer support, which is the provision of support to an individual engaged in delinquent behavior by someone who has previously also practiced such behavior, is one such application. Little is known, however, about its effects on desistance, or the mechanisms that explain these effects. On the basis of theoretical papers, program descriptions, and interviews with experts, we developed an initial program theory. We propose seven mechanisms that might play a role in the (potential) effects of support by experiential peers: (1) empathy and acceptance, (2) social learning, (3) social bonding, (4) social control, (5) narrative and identity formation, (6) hope and perspective, and (7) translation and connection. In addition, in this protocol paper, we describe the methods of a systematic realist literature review that will be conducted in order to investigate the evidence base for this program theory.

**Methods:**

The study described in this protocol paper is a realist review, which is a suitable approach to study complex interventions and fits the explanatory purpose of the study. We outline the steps to be taken for the systematic realist review, including the selection and assessment of studies and the methods for synthesizing the findings.

**Discussion:**

Investigating the effects and the underlying mechanisms of support by experiential peers for individuals with delinquent behavior is relevant because the forensic setting has some unique features, and the involvement of service users might create even more tension than in other settings due to stigma and perceived risks. The findings that will be reported in the realist review will contribute to the knowledge of the effects of support by experiential peers and will provide insight into which aspects remain to be studied. It might also provide formal care institutions with guidance on whether to involve experiential peers in the delivery of their services and the conditions under which these interventions are likely to lead to positive results.

**Electronic supplementary material:**

The online version of this article (10.1186/s13643-019-1036-2) contains supplementary material, which is available to authorized users.

## Background

Individuals who have demonstrated delinquent behavior tend to be considered a difficult-to-reach population, due to the partly concealed nature of their behavior and their rejection of help. Criminal participation peaks during late childhood and adolescence, and most individuals tend to desist from delinquent behavior in early adulthood [1]. However, longitudinal studies show multiple trajectories [2], indicating that although this behavior is largely normative and limited to adolescence, for a part of the population, the involvement in delinquent behavior is more severe and persistent. For these individuals, desistance might not be such an obvious development. It is therefore relevant to investigate interventions developed to stimulate, accelerate, or support this desistance process.

However, previous studies have found that adolescents and emerging adults with delinquent behavior display an excessive need for self-reliance, which forms a barrier to care utilization [3]. This might partly consist of a “normal” need for autonomy during maturation into adulthood, and, with increasing age, some of these adolescents might become more inclined towards desistance, which they tackle on their own or seek assistance for. However, the reluctance to seek or accept help might be more persistent for people who hold negative attitudes and beliefs towards (mental health) help-seeking, based on previous experiences (e.g., feeling that they had not been taken seriously) [4]. This might also be true of those who experience a fear of stigma, including from the person who is providing the help [5]. In a study on at-risk adolescents and emerging adults, the interviewees indicated that they did not want any help, since they felt that others did not understand them, especially when they had not been through similar experiences [6, 7]. The formal care system is overrepresented by highly educated people [8], who in most cases do not have any personal experience of delinquent behavior or even with growing up in a criminogenic environment. This does not imply that professional care providers without such experiences lack the capacity to help or support people who present with delinquent behavior. Among this group are many experienced practitioners who have the necessary skills and features to connect with the target population and to contribute to behavioral change. A discrepancy in personal background between client and practitioner might even create opportunities for clients to become acquainted to a different kind of world and as such provide opportunities to increase their bridging capital [9]. However, for a part of the target population, this dissimilarity may result in not accepting professional help or care because they perceive or assume a mismatch between their own personal characteristics and life experiences and those of the professional practitioner. This (mis)match can be highly relevant, because studies have shown that the relationship or working alliance between a client and practitioner (whether a therapist or a probation officer) plays an important role in achieving behavioral change [10]. Investigating the potential benefits of support provided by people who have a background similar to individuals who engage in delinquent behavior is therefore an important undertaking, because it might aid us to gain a better understanding of what works for them and under what conditions. The main purpose of the study described in this protocol paper is thus to investigate the effects of support by experiential peers on desistance and related outcomes and to provide insight into the mechanisms involved, as well as the contextual factors that affect these mechanisms.

The concept of “experiential expertise” is increasingly being implemented in mental healthcare [11]. Specifically, in mental health services with a recovery orientation, the involvement of clients has become essential [12], which makes it likely that the field of criminal (juvenile) justice will follow suit. The perspectives of service users are increasingly being recognized as important in the process of designing and implementing interventions. Listening to their needs can help practitioners to develop approaches that are perceived as more meaningful and supportive of processes of change [13]. According to McNeill [14], service providers who aim to affect the rehabilitation of offenders should come to see themselves more as supporters of the desistance process, of which the offender is the owner, rather than as providers of correctional treatment belonging to the authorities. A more direct way in which experiential expertise is mobilized is by letting former service users serve as peers, directly providing support and guidance to current clients or patients. This is the type of application of experiential expertise that will be central to the review described in this protocol. From here on, we will refer to this as “experiential peer support” or “support by experiential peers.” This phrase does not include naturally-occurring relationships between people with similar experiences and does not take into account whether someone has had any formal training. Since we are aware that having certain experiences does not necessarily qualify someone to provide support to others with complex problems, in the realist review, we will make a distinction between levels of expertise in order to take into account the effects of formal training.

Most research on the effects of experiential peer support has been conducted in a mental health services setting. In their review, Repper and Carter [15] found some studies that report positive results of experiential peer support with respect to relapse rates, empowerment, social functioning, and mental health. For the studies that found no difference between peer and non-peer staff, they concluded that this “demonstrates that people in recovery are able to offer support that maintains admission rates (relapse rates) at a comparable level to professionally trained staff” [15]. Although these results might also be valid for the forensic setting to some extent, it remains relevant to study the mechanisms specifically in this setting, because it has several unique aspects. Firstly, according to South, Bagnall, and Woodall [16], even though individuals presenting with delinquent behavior might be more open to advise and support coming from peers, their resistance to authority might still cause them to resist this opportunity. In addition, peers meant to support the receiver in the process of desistance or rehabilitation might in fact support risky behaviors [16]. This could lead to deviancy training, which is an adverse (iatrogenic) effect that can occur when deviant peers are aggregated, leading to an increase of problem behavior [17]. This risk emphasizes the necessity for an evidence base for such types of intervention. Lastly, it is likely that stigma and prejudice among professional care providers are even more strongly present and persistent regarding ex-offenders than for experiential peers in other fields of (mental health) care. This makes it a more precarious situation, in which sufficient attention should be paid to the implementation of the intervention and the conditions that could increase the chances of success. Bagnall et al. [18] conducted a systematic review of peer support in prisons, which showed that such services had a positive effect on recipients of this support, emotionally and/or practically. However, it is also relevant to take into account other settings, because not all individuals who display delinquent behavior are sentenced to imprisonment. This holds true particularly for adolescents. In addition, this type of support might be as effective or even more effective in other settings, such as when the individual is under probation or when he or she is released from prison, and working on rehabilitation and reintegration. To the best of our knowledge, this is the first systematic realist review of the effects of support by experiential peers on desistance and desistance-related outcomes in which there is an explicit emphasis on the mechanisms and contextual factors that play a role in the effect of these types of interventions. With our review, we hope to contribute to the third generation of research on the subject of peer support (the first two stages involved feasibility studies and studies of peer staff in conventional roles), which, among other things, poses questions concerning the unique aspects of support by experiential peers, the outcomes they might produce, and the active ingredients responsible [19].

In this protocol paper, we will describe the concept of experiential peer support and the types of interventions related to it. Furthermore, following a realist approach, we will present our initial program theory and describe the mechanisms proposed to play a role in the effects of support by experiential peers on desistance and desistance-related outcomes. Lastly, we will describe the methods of our realist review and provide an overview of the steps that we will take to conduct this review.

### Experiential peer support

Interventions for people who engage in delinquent behavior provided by people with experiential expertise can serve several functions. For a systematic review of peer interventions aimed at improving health in prison settings, a typology was developed of the various forms that peer-based interventions can take [16]. Those most relevant for our review appear to be *peer support* (providing emotional or social support, or practical aid), *peer mentors* (role models who establish a supportive relationship with their mentee), and *peer workers* (providing informational support and connecting individuals to services in the area of health or welfare). *Peer education* and *peer training* seem to have a more instrumental and didactic focus, and it is unclear to what extent there is room for a relationship to develop between the peer educator or trainer and the recipient. In practice, however, the lines between the types of interventions become blurred. Experiential peers might take on several different roles at once or might progress from one role to the next as the relationship evolves.

Despite differences in goals and tasks, interventions involving experiential peers have in common that their core is the principle of homophily or the idea that people are more likely to connect with people similar to themselves [20]. In order to achieve social goals, such interventions involve the use of the communicative and social mechanisms that occur between people with similar experiences [16]. Individuals might share elements of a similar reality and a common language [21]. They might also share similar experiences, including “having been through a condition and handling multiple problems, having lived through treatment, the social consequences of a condition (stigma) or the experience of discovering a coping strategy within oneself” [22]. It is particularly important when providing support to someone going through a status transition, such as the transition from “offender” to “ex-offender,” that one has experienced a similar transition [23]. For the purpose of studying the effects of peer interventions targeted at a justice-involved population, we are mostly interested in those interventions in which the provider of the support, the experiential peer, has already experienced this transition and is thus further along the desistance process. In the review, we will therefore focus on experiential peer support as involving an “asymmetrical relationship, with at least 1 designated service/support provider and 1 designated service/support recipient” [24].

### Initial program theory

As part of the initial program theory, in which the experiential peer support intervention is the “program,” seven mechanisms will be presented. These are hypothesized to play a role in peer support interventions, eventually leading to one or more of the desired outcomes regarding the process of desistance. We have not undertaken an attempt to construct specific context-mechanism-outcome (CMO) configurations at this stage and therefore will not make any claims regarding specific relationships between what we consider contextual factors, mechanisms, and outcomes. In order to construct this initial program theory, we used non-empirical articles found in preliminary searches in the initial stages of the review. Our sources included theoretical sections of reviews, program descriptions, and descriptive papers on the utilization of experiential expertise in the support of individuals engaged in delinquent behavior or with other problems. Insights from criminological and psychological theories were also used to substantiate the assumed link between mechanisms and outcomes.

In addition, the first author (ML) conducted semi-structured interviews, lasting between 62 and 97 min, with four individuals who have expertise in the subject matter. The first interviewee is an expert in the field of role models for juvenile delinquents. The second interviewee has experiential expertise in mental healthcare and the third in the forensic mental healthcare. Both use their expertise in their current positions and are well-known experts in the field of experiential expertise. The fourth interviewee is a former offender who is now working as a formal care provider. All four interviewees were approached through e-mail. The first three interviews took place at the interviewees’ offices; the fourth interview took place at the interviewee’s home. The main topics of these interviews were general opinions on support by experiential peers for the target population of individuals with delinquent behavior, the potential benefits/effects and risks, potential mechanisms, and contextual factors that influence the effects of such interventions. If certain aspects (e.g., timing, requirements regarding the experiential peer) were not mentioned spontaneously, the interviewees were asked specifically to reflect on these. Based on the first interview and the literature, a preliminary version of the model was constructed. This model was presented to the second, third, and fourth interviewee. The input of the interviewees was integrated into the description of the model and can be found in Table 1 (mechanisms) and Table 2 (contextual factors). Throughout the descriptions of the different mechanisms and contextual factors, the same tables can be consulted when referring to the interviewees. The interviewees were given the opportunity to check whether their input was correctly represented in this protocol paper and to give feedback prior to its submission.

**Table 1:** Elements mentioned by interviewees

Mechanism	Important elements according to interviewees
Empathy and acceptance	The experiential peer is not judgmental^1,3,4^; shows positive regard for the recipient^1^; is not occupied with truth-seeking^3^, and sees the recipient as an equal^3,4^
Social learning	The recipient might learn to deal with criminogenic factors^1^, build resilience against negative imaging and stigmas^3^, and acquire the wish to also contribute to society^2^. The experiential peer might help the individual to make sure that his or her survival behavior is not carried over into the outside world^3^.
Social bonding	The relationship with the experiential peer might be a trusting relationship^4^; the experiential peer might help with closure of former (negative or damaged) relationships and dealing with this grief^1,3^
Social control	The experiential peer might be quicker to see through the client’s motives^1^, might feel more comfortable correcting the client^1,4^, and might be able to ask critical questions^3^
Narrative and identity formation	Through the support of an experiential peer, the recipient might be empowered (related to their identity)^1,4^, embrace the past^2^, complete his or her narrative^3^, and gain a sense of agency^3^
Hope and perspective	The experiential peer might provide hope^2,3,4^, might enable the individual to envision an alternative future^1,3^, and might be someone who believes in the individual^3^
Translation and connection	The experiential peer might form a connection between the individual and formal care^1,3^ and might translate between the client and formal care^1^

^1^Lector juvenile delinquency and researcher; ^2^Experiential peer (mental health care) and researcher; ^3^Experiential peer (forensic mental health care) and trainer, ^4^Experiential peer (no training) and formal care provider

**Table 2:** Contextual factors mentioned by interviewees

Contextual factor	Important elements according to interviewees
Timing	Support by an experiential peer might be beneficial in various stages: before something occurs, when there are already some signals, when something has already occurred, and during aftercare or rehabilitation^3^; probably the sooner the better^1^; and the individual should be willing to take steps towards desistance^1^
Prerequisites of experiential peer	Experiential peers should - be credible^1^ and realistic^1^ - be respected by the client^1,4^ (by having displayed criminal behavior of similar severity^4^) - be willing to learn about methodological and evidence-based practices^1^ - learn how to navigate in a system with political interests and bureaucratic restrictions^1^ - be able to reflect on own experiences and integrate these with those of others^2^; know what has and has not helped them and that this might be different for someone else^2^; and be capable of self-reflection^3^ - not have a distancing attitude^2^ - be approximately the same age as the client^2^ - if applicable: have been released from prison some time ago^3^ - know the difference between utilizing own experiences and glorifying them^1,2,3^ - focus on the client’s story and adapt their support to that^3^ - not be too radical in their rejection of “the system” or society^1,4^

^1^Lector juvenile delinquency and researcher; ^2^Experiential peer (mental health care) and researcher; ^3^Experiential peer (forensic mental health care) and trainer, ^4^Experiential peer (no training) and formal care provider

### Outcomes

We have chosen to interpret desistance as a broad concept rather than focusing on refraining from offending as the sole outcome. We made this choice for several reasons. Firstly, we aim to follow the recent emergence of positive criminology, in which the focus is on resilience and rehabilitation rather than on solely quitting criminal behavior [25]. Secondly, we consider desistance to be a process rather than a clear endpoint, encompassing a complex interaction of subjective and social factors [26]. Nugent and Schinkel [27] propose a terminology for the various types of desistance, which is based on the distinction by Maruna and Farrall (2004, as cited in [27]), namely primary and secondary desistance, and the addition of tertiary desistance by McNeill (2016, as cited in [27]), but which does not suggest an order in time or importance. *Act-desistance* here refers to refraining from offending, *identity desistance* describes the internalization of a new identity as a non-offender, and *relational desistance* concerns the recognition of change by others at the micro, meso, and macro levels [27]. In addition to these types of desistance, we will also consider several other outcomes that are not easily categorized, namely increased social capital, positive personal development, improved mental health, and positive changes in personal circumstances, such as employment.

**Fig. 1:**
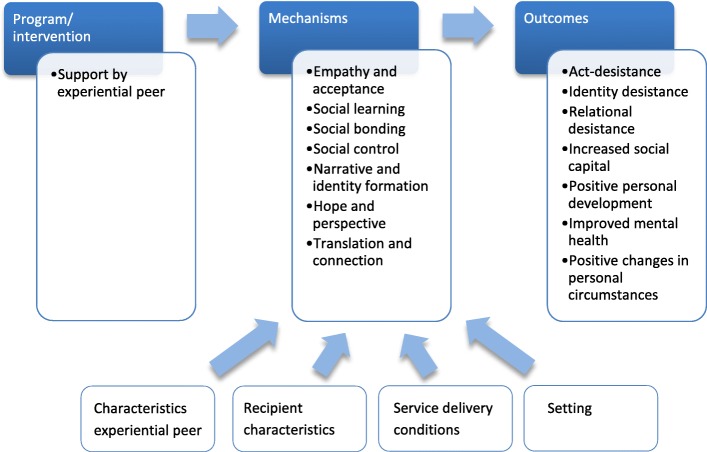
Graphic representation of the initial program theory

The mechanisms that will be proposed as explanations for how desistance might be achieved through support by experiential peers are likely to fall under one of three categories of theoretical explanations, as distinguished by Maruna [28]. The first category includes the so-called *ontogenic theories* that focus on maturational reform, or the idea that offenders “grow out” of their delinquent behavior as they become older. This is not merely a passive process of becoming biologically older, as it is interpreted by some, but should rather be seen as a feeling of becoming “too old” for certain types of behavior, after which the individual takes steps to break with (friends and lifestyles of) the past, develops new routines, and settles for a less “exciting” life [29]. *Sociogenic theories*, which constitute the second category, are concerned with the importance of social bonds in explaining changes in delinquent behavior across the lifespan. The third category comprises *narrative theories*. These theories stress the importance of subjective alterations in a person’s sense of self and identity, which in turn are reflected in motivation, a greater concern for the future, and more consideration for others [28]. In conclusion, there is a broad range of desired outcomes, from reduced involvement in delinquent behavior to positive personal development and improved personal circumstances (see Fig. 1).

### Mechanisms

Based on the literature and the interviews, we propose seven main mechanisms through which interventions involving experiential peer support might lead to desistance-related outcomes. For some of the mechanisms, the emphasis is on the role of the experiential peer, whereas for others, it is more about how the receiver of the intervention reacts. However, for all the mechanisms, the key is the interaction between the two actors. The mechanisms overlap to some extent and are not expected to operate in isolation from each other. There might be interactions between the mechanisms and between the outcomes, and the outcomes might in turn also influence the mechanisms. In addition, some of the proposed mechanisms might also be valid for general peer support interventions or support structures in other settings. In this review, however, we will focus on what it is about receiving support from an *experiential peer*, with shared experiences of *involvement in delinquent behavior* that might make these mechanisms particularly relevant. An overview of the mechanisms is provided in Fig. 1.

#### Empathy and acceptance

Firstly, it is proposed that experiential peers, due to their background of similar experiences, might be more capable of experiencing empathy for others who engage in delinquent behavior and of accepting them. In addition, according to Carl Rogers, genuine empathy and unconditional positive regard for the client are necessary conditions for personality change, such as moving from immature behaviors towards behaviors that are considered more mature [30]. Kindness and emotional support promote confidence and the feeling that one matters [31], thus increasing one’s sense of self-worth and self-esteem. Empathy for someone with delinquent behavior might be easier to achieve if one has lived through similar experiences [18, 32]. An important aspect mentioned to some extent by interviewees 2, 3, and 4 is that the experiential peer knows what it is like to live with the same feelings of pain and distress that the individual is experiencing. In an institutionalized setting, for instance, the experiential peer knows that tension builds up prior to important (treatment) appointments and is also familiar with the situation of confinement that one has to return to afterwards. From their own experiences, experiential peers might be better able to understand these feelings and support the individual in processing these. When desistance has already been initiated, experiential peers might play a supportive role in its maintenance, which could be accompanied by the “pains of desistance,” such as the pain of isolation, the pain of goal failure, and the pain of hopelessness [27]. Similar others might not only be considered better equipped to support individuals in distress in terms of understanding their feelings; they are also less likely to reject someone because he or she is distressed [23]. This was also mentioned by interviewees 1, 3, and 4, who believe that experiential peers can make individuals feel that they are important and not looked down upon nor judged for their actions. While the individual might face stigma, exclusion, and skepticism from others, the experiential peer will offer acceptance and inclusion [21].

#### Social learning

Through the second mechanism, social learning, the individual might learn behaviors, skills, or attitudes that will support him or her in the process of desistance. It is argued that the individual learns in interaction with the experiential peer and by general social learning mechanisms, such as imitation and differential reinforcement. This is similar to the ways in which delinquent and deviant behavior is learned, according to Akers’ social learning theory [33]. The content of what is modeled and learned might encompass ways in which the experiential peer is able to refrain from offending, effective problem-solving strategies, and useful skills necessary for dealing with (psychological, social, and financial) challenges related to reentry [34]. Two such challenges are not succumbing to peer pressure without losing respect and resisting tempting opportunities to acquire money illegally [35]. These skills are extremely important, because young offenders returning to their community find it undesirable and sometimes even impossible to isolate themselves from their “negative peers.” Moreover, the opportunities and assets resulting from delinquent activities might still have an appeal for them [36]. The desire to desist, which might not be inherently present in the individual, is another aspect that can be mimicked [21]. In addition, the experiential peer might support the individual in the process of abandoning certain beliefs, attitudes, and behaviors that were once learned as survival mechanisms in settings such as prisons [34]. Lastly, experiential peers might transfer knowledge to the individuals and provide them with advice or guidance on how to deal with the justice system and the conditions, requirements, and obligations that come with it. Peers might be considered more credible role models or sources of knowledge than staff due to their personal experiences [18, 32]. Credibility is considered an important factor that influences the extent to which modeled behavior will be imitated [37]. An experiential peer is also more likely to be seen as a realistic role model. This is crucial, since, as interviewee 1 explained, it is not recommended to present role models who did not have to deal with similar stressors in life, because most adolescents who engage in delinquent behavior growing up in disadvantaged situations will not be able to achieve a similar status to such role models. It is also assumed that other conditions for successfully adopting the behavior, such as having opportunities to practice the behavior and reinforcement of this newly-learned behavior [37], will be met in the ongoing relationship with an experiential peer. Lastly, having a positive role model might negate the influence of negative role models, such as friends or siblings who are involved in delinquent behavior [22].

#### Social bonding

The third mechanism is the development of a social relationship. An individual with delinquent behavior might find it easier to trust a peer than professionals [18], and deeper levels of similarity (attitudes, beliefs, values, personality) between individuals and experiential peers might be related to a higher quality relationship [38]. Furthermore, disclosure on the part of the peer might stimulate more disclosure by the individual, possibly leading to the development of a more meaningful and close relationship [39, 40]. Adolescents or emerging adults in particular might also be more able to establish positive social bonds with others due to a positive experience and regained trust in adults [41]. Also, as interviewees 1 and 3 explained, the individual might, with the help of an experiential peer, become better at finding closure in relationships that are not supportive of their desistance process. This does not only apply to relationships with deviant friends, but rather to damaged relationships in which the individual is still emotionally invested and which deter him or her from moving on. The resulting higher quality of social bonds might lead to desistance in several ways. According to Laub and Sampson [42], as social capital increases, the individual is equipped with more resources for support and problem solution. Simultaneously, there is more at stake and less time, making criminal activities less attractive and opportune. Although the individual does not have complete control over what happens at the social level, he or she does exercise human agency and can either seize opportunities that could become turning points, or ignore them [42]. Matching an individual with delinquent behavior to an experiential peer might thus present an opportunity for a new relationship that can provide support and problem-solving skills.

#### Social control

The fourth mechanism that will be discussed is social control. Borrowing this term from the field of health psychology, it here refers to interactions within personal relationships that involve influence and regulation [43]. Social control might operate indirectly, for example when the individual has internalized a feeling of accountability towards the experiential peer, and therefore avoids deviant behavior. Our interviewees, however, seemed to refer more to the direct type of social control in which someone motivates or urges an individual to quit negative behaviors or to engage in positive behavior [43]. Experiential peers might recognize former own attitudes or behaviors in the person they are trying to support and will therefore be better able to see through a socially desirable act by the individual and be quicker to ask critical questions, as interviewees 1 and 3 explained. The individual might also be more sensitive to corrections coming from someone with similar experiences. This idea, namely that experiential peers might be quicker to act to convince an individual to quit negative behavior, might also be because they are more likely than a professional care provider to anticipate challenges related to reentry, address these, and respond to them in order to prevent escalation or relapse [34]. In addition, the experiential peer, who probably has more time and flexibility, is able to monitor the process of desistance and to detect any risks of re-offending [35].

#### Narrative and identity formation

The fifth mechanism, “narrative and identity formation,” denotes the process of the formation of a new identity, including the self-narrative regarding someone’s criminal justice involvement. It is related to the first mechanism, but the focus is more on self-acceptance rather than that of others. According to Maruna [28], desisters differ from those who persist in crime by their self-narratives. Those who refrain from offending, presenting *redemption scripts* instead of *condemnation scripts*, tend to take responsibility for their past behavior and make a deliberate effort to abandon a life of crime [28]. This suggests that the key to desistance is not hiding or disregarding experiences of delinquent behavior, but rather incorporating these into one’s multi-faceted identity or personal history. An experiential peer might model such an identity and provide opportunities for new roles to be practiced [21]. As interviewees 1 and 4 indicated, identity is extremely important to this population. When individuals with delinquent behavior see that someone with a similar background who now has a “regular” job is not necessarily a “loser,” this might open up opportunities to them to maintain several aspects of their identity that were previously related to their status as an offender. Furthermore, whereas in other environments individuals might find their versions of reality degraded by others, the bond with another who shares a common experience allows space for marginalized perspectives and might even lead to a sense of empowerment [21]. By believing in the individuals’ abilities, which are part of the new role, they furthermore realize that they are worth something and start to believe in themselves as well [28].

#### Hope and perspective

Experiential peers, as credible and valuable models of the idea that recovery is attainable, might furthermore instill hope and provide perspective for individuals with who engage in delinquent behavior, which is the sixth mechanism that was mentioned in the literature [34, 44, 45], as well as by most interviewees. Seeing that others who have experienced similar situations have been able to get through them might be inspirational to those still finding their way. A significant other might not only help them to envision an alternative identity, but also an alternative future [28]. According to LeBel et al. [26], hope is not only about wishing that something will change, but also entails the perceived availability of ways to achieve these goals. They find that hope, or the belief in self-efficacy, “may be a necessary if not sufficient condition for an individual to be able to desist from crime” [26]. Interviewee 3 indicated that this might be because hope leads to an increase in motivation, which stimulates the individual to actually take steps to benefit from support. Also, compared to persistent offenders, desisting offenders tend to have a stronger sense of agency [26], which, according to Maruna [28], is a prerequisite for resisting and overcoming structural criminogenic factors. Altogether, the individual might be more motivated to change certain aspects of his or her life, have a stronger sense of self-efficacy (since someone coming from a situation similar to theirs was also able to achieve desistance), and feel more empowered. If the individual additionally gains a sense of agency and responsibility, there is an increased likelihood that he or she will undertake steps to refrain from offending.

#### Translation and connection

The seventh and last mechanism that is hypothesized to play a role in the effect of experiential peer support on desistance-related outcomes is the bridging position of experiential peers. The latter speak the same language as the recipients and know their living environment, but are also familiar with the world of formal care and the justice system. The experiential peer might play a role in translating the social world to the individual, which might refer to translating professional speech into everyday language, but also to explaining the requirements of society to be included to those who may have been physically excluded from it [21]. Moreover, the experiential peer, in contact with formal care, might speak on behalf of individuals and advocate for them. In addition, if a trustful relationship has been built between the individual and his or her mentor, the individual might be more likely to be open to seeking or accepting help [31]. The trust on which this relationship is built thus helps to link the individual to treatment and services, and experiential peers are able to help the individuals to utilize these services and support them in this engagement [34, 35]. However, as interviewees 2 and 3 emphasized, it is crucial that individuals continue to have an agency with respect to which resources they want to utilize. It should not be assumed that utilization of care is necessarily a positive thing, because if this care is not suitable for the individual, it could have detrimental effects. The experiential peer might further link individuals to educational, housing, or vocational opportunities; advise them in these areas; and accompany them to important appointments [34]. It can be argued that an experiential peer thus contributes to the linking social capital the individual has, which refers to relationships that connect people across “‘vertical’ power differentials, particularly as it pertains to accessing public and private services” [46]. The relationship the recipients have with an experiential peer can therefore be seen as one that connects them to opportunities that might be able to help them get ahead.

### Context

Several contextual factors might influence whether the mechanisms are activated and thus whether the likelihood of desistance is increased by providing peer support by an experiential peer. In addition to consulting the literature, we spoke to our interviewees about conditions for the successful implementation of such peer support. These can be found in Table 2. Firstly, it is conceivable that the characteristics of both the individual and the experiential peer might alter mechanisms. Experiential peers might need to possess a certain level of maturity and experience [32]. Interviewees 2 and 3 mentioned that it might be important that experiential peers have not been involved in delinquent behavior for a considerable amount of time in order to prevent any glorification of criminality. Some distance (in time and in attitude) towards their criminal career might also counter the risk of deviancy training. Interviewee 4, in addition, mentioned that in order for experiential peers to be taken seriously by the individual and to be respected in their roles as experiential peers, the level of criminal behavior they were involved needed to be “severe enough.” On the receiving end of the intervention, younger individuals or those with more severe criminal careers might respond differently to peer support. The interviewees did not indicate an ideal timeframe in which the individual might be most susceptible. According to interviewee 3, experiential peer support is most important during rehabilitation or aftercare, and interviewee 1 indicated that the individual should at least be ready to take steps towards desistance. Service delivery conditions might be influential as well. The careful implementation of peer support might increase acceptance by professional staff and thereby improve embedding in and cooperation with formal care. Proper recruitment, training, and support of experiential peers [32], which is considered important by interviewees 1, 2, and 3, might help them to reflect on their own experiences, integrate them with those of others, and gain an understanding that what helped them might not work for someone else. Lastly, the setting of the intervention might play a role. Experiential peer support might be part of a program in prison, but it might also be offered within a mental healthcare facility or as a voluntary service. The function and security level of the facility in which the intervention is offered might therefore affect its success [32].

### Aim of the review

Through the realist review approach, the study described in this protocol paper will comprise an investigation of the effects of support by experiential peers on desistance and desistance-related outcomes, with the aim of providing insight into the mechanisms involved and the contextual factors that affect these mechanisms.

### Review questions

1. What is the effect of support by experiential peers for individuals who engage in delinquent behavior on desistance or desistance-supportive outcomes?
2. What are the mechanisms involved in these effects?
3. Which contextual factors have an influence on the mechanisms or outcomes?

## Methods

### Realist review

For our review, we will be mainly following the realist review processes as described by Pawson [47] and the Realist and Meta-Review Evidence Synthesis: Evolving Standards (RAMESES) guidelines as described by Wong and colleagues [48, 49]. The approach of realist review or realist synthesis was chosen because it fits the explanatory purpose of the review. While a traditional systematic review might provide evidence on whether an intervention is effective, it does not always provide insight into how or why it might work or how it is influenced by contextual factors. Furthermore, a realist review is a suitable approach to study complex interventions [50]. Experiential peer support involves the development of a social relationship between two human beings. In addition, this is not a naturally occurring relationship, but one that operates within a formal setting. This is a complex matter: it is about more than merely putting two people together. What happens in this relationship, and whether an actual relationship develops at all, might depend on many factors. As Wong et al. [48] point out, what might trigger change is not the intervention itself, but rather how the participants react to the opportunities created by it. In our study, the opportunity to build a trusting relationship or to learn from another person’s experiences might lead participants to think differently about their experiences and see other alternatives. A realist approach allows for testing multiple mechanisms through which these interactions might contribute to desistance. Furthermore, this approach takes into account the context that might influence the mechanisms, such as participant characteristics (of both provider and recipient of the intervention), service delivery conditions, setting, and geographical location.

In the introduction of this protocol paper, the initial program theory, including proposed mechanisms and contextual factors, was described. In the following, we outline the search strategy and selection procedure that was utilized to source relevant studies, which will serve to refine the initial program theory in order to provide an evidence-based explanation for the potential effectiveness of experiential peer support. The protocol is described following the PRISMA-P checklist [51, 52], added as Additional file 1. The protocol functions as a guideline, because realist reviewing allows for an iterative approach in which the activities can be tailored to the available findings.

### Study inclusion criteria

Studies will be included if they were published between 1990 and 2018 in English-language journals and if they fulfill the inclusion criteria described below.

#### Population

We will include studies involving individuals who have displayed delinquent behavior in the past or are still involved in delinquent activities, and who are receiving or have received an intervention involving experiential peer support. The use of illicit drugs or involvement in sex work is not considered a delinquent behavior in this study.

#### Intervention

Studies will be included if experiential peer support is a central element of the intervention or the intervention has a different central element (training, therapy, probation service) but is led by a peer or makes use of the difference in experiential knowledge between participants. Although it might not always be clear whether the experiential peer is a step further in the desistance process, we will aim for these types of interventions by only including those that involve asymmetrical relationships in which there is a clear role distinction between the person providing the support and the person receiving the support. This means that mutual help groups or supportive communities in which participants have equal positions and are simultaneously a receiver and provider of support will be excluded. Studies will be included when the intervention is aimed at achieving desistance or desistance-supportive outcomes for the person receiving the support. These outcomes include but are not limited to:

- Positive changes in delinquent behavior (e.g., abstinence, lower frequency, less severe types of crimes)
- Increased social network (e.g., more social bonds with others or society, higher quality relationships, increased social networks, and voluntary utilization of suitable resources)
- Positive personal development (e.g., coping skills, self-efficacy, self-esteem, future orientation, problem-solving skills)
- Positive changes in personal circumstances (e.g., employment, education, housing)
- Improved mental health (e.g., decrease in symptoms, substance abuse)

Interventions aimed at improving participants’ physical health will not be included.

#### Types of study

There will be no restrictions based on methodology: all types of designs, quantitative and qualitative, can be included. The reviewed studies should, however, be empirical and have gathered data on the outcomes of the intervention, mechanisms, or contextual factors that play a role. Although we exclude documents that do not contain empirical data from the review itself, we have made use of several theoretical pieces in the development of our initial program theory. In addition, when analyzing the data, we will not only look at the outcomes of the study, but also take into account the background and introduction sections of documents, as these might contain relevant information on how the intervention is expected to work or on why it did not work as expected. This information can then be compared to data found within other documents [53].

### Search strategy

The initial systematic literature search was carried out on July 30, 2018, using eight electronic databases: Embase, MEDLINE, PsycINFO, Web of Science, Scopus, Criminal Justice Abstracts, SocINDEX, and Google Scholar. The complete search strategy can be found in Additional file 2. Its content was determined by the first author (ML) in consultation with the second (FJL) and last author (GEN). The technical construction of the search strategy was done together with two information specialists of the Erasmus University Rotterdam over a period of 2 months during which several meetings took place in which the search was piloted and refined. The final search consisted of three elements, with the first part covering the target group (using keywords such as “delinquent behavior,” “crime,” and “offender”), the second part being related to the setting of intervention (e.g., “probation,” “detention,” and “mental healthcare”), and the third part aimed at selecting papers in which an intervention involving experiential peers was investigated (e.g., “peer support,” “self-help group,” and “experience expert”). For this search, no proposed mechanisms or outcomes were specified in order to not exclude any unforeseen elements. Furthermore, the search strategy did not have any methodological filters, as is common for realist reviews [48]. This first search yielded 7976 results, with 4867 unique results after deduplication.

After the evaluation of the results from the first search, an additional search might be done in order to refine several elements of the program theory, as is common for realist reviews. For instance, if insufficient information is found in the initial search regarding on one or more of the mechanisms or contextual factors, this second search will serve to find relevant studies investigating these aspects in other domains, because these studies might still empirically support the program theory. An upgrade of the search will be done before publishing the review. If necessary, this search will contain additional keywords that were found in the literature. Other methods for identifying relevant research might be used, such as reference checking and hand searching of these, which is as much used as conventional database searching in realist reviews [50].

### Selection of studies

In order to make a selection out of the 4867 results, all titles and abstracts were read and reviewed for inclusion in light of the abovementioned inclusion criteria. This was done by two reviewers (ML and TM) using a fast, independent method for categorizing abstracts as “Includes” or “Excludes” in EndNote [54]. Using this method, both researchers read all abstracts and dragged articles to the custom groups “Includes” and “Excludes” corresponding to their verdicts. The included references of both reviewers were then combined into one library. Duplicate references, which have been included by both reviewers, were selected for full-text review. The nonduplicate references, those for which there was no initial consensus, were discussed until an agreement is reached. This first selection consisted of 130 articles. The next step will be to scan the full-texts of the “Includes,” again focusing on the inclusion criteria. This will be done prior to extensively reading the articles, since it is expected that this first selection will contain a large amount of noise, because the titles and abstracts do not all contain a sufficiently detailed description of the intervention. Again, this will be done by two reviewers (ML and a research assistant) independently and, in case of any disagreement, the papers will be discussed. When necessary, a third researcher (GEN) will be involved. Depending on the quantity and quality of the findings after evaluation of the full texts, the final selection for analysis and synthesis might be restricted to:

- A target population of adolescents and emerging adults (e.g., ≤ 30 years old)
- Delinquent behavior that is not domestic abuse, intimate partner violence, or DUI-offenses
- One-on-one interventions

Restricting the study to a certain age category (in which participation in delinquent behavior is highest) allows for a more homogenous study sample. If possible, we will exclude articles that are focused only on domestic abuse, intimate partner violence, and DUI-offenses. We consider these types of offenses to be of a distinct category with other underlying factors. Lastly, we are mostly interested in one-on-one interventions, since these provide the clearest opportunity for real relationships to develop between the providers and the recipients.

### Data extraction

The data extraction will consist of two procedures. Firstly, a research assistant will register document characteristics and study details into an Excel spreadsheet. This spreadsheet consists of several components: (1) general information regarding the document, such as the year and country of publication, study funding, and potential conflicts of interest; (2) general information regarding the study, such as the study design, population, duration, and setting; (3) information regarding the participants, such as the method of recruitment, inclusion and exclusion criteria, and the size and composition of the final sample (e.g., age, gender, ethnic background); and (4) information regarding the type of intervention, such as whether the experiential peer support was a standalone intervention or part of a larger program, and the characteristics of the experiential peers included in the study. Ten percent of this part of the data extraction will also be done by the first author (ML) to check for consistency. The second part of the data extraction will consist of coding the included documents using the software program NVivo. This step is meant to provide an overview of the information in the documents regarding our research questions concerning mechanisms, outcomes, and contextual factors. We will make use of deductive and inductive coding. For deductive coding, we will use codes created in advance reflecting the mechanisms, outcomes, and contextual factors we have proposed in our initial program theory. In addition, with inductive coding, we have the opportunity of adding codes that originate from the data, such as mechanisms or potential (positive or negative) outcomes that were not included in our initial program theory. The coding will be done by the first author (ML) and 10% of the documents will be coded by the research assistant in order to check for consistency. If this consistency turns out to be satisfactory, this quality control check will be sufficient. Any disagreements will be discussed and if necessary, a third researcher (GEN) will be consulted.

### Quality assessment

Next, the included papers will be assessed by two reviewers (ML and GEN) on two aspects: relevance and rigor. Articles will be more likely to contribute to the refinement of the initial program theory if the methods used to generate the relevant data are considered suitable and credible. It is, however, important to realize that there does not need to be a relation between the rigor and the relevance of the data [53]. For instance, a document may contain very relevant information on a relationship between a mechanism and an outcome even if this is not what was empirically tested in that specific study. In such cases, we might want to zoom in on that particular relationship in other documents or in an additional search, in order to find data that is more rigorous or trustworthy.

In order to evaluate the rigor of studies, we will use the data extraction spreadsheet. The main question for this part of the analysis is whether the data are sufficiently trustworthy and credible to justify changing or corroborating (parts of) the initial program theory. Quantitative studies will be assessed on study design, sample size, participant selection, operationalization of outcomes and mechanisms, and adjustment for confounders. For qualitative studies, the assessment will be based on participant selection, the extent to which data collection and analysis are described, the operationalization of outcomes and mechanisms, and the credibility of the findings. In order to evaluate whether sections of the documents are relevant to the development of our program theory, we will use the references that were coded with NVivo. For all coded sections, we will evaluate whether they describe an element of the program theory that we aim to refine. Sections of the documents might refer to mechanisms, outcomes, and contextual factors that were already included in the initial program theory. They might also contain relevant information on specific CMO configurations or unforeseen, additional mechanisms, contextual factors, or outcomes. The two reviewers will score aspects of relevance and rigor as low, moderate, or high using a codebook based on that used by Nagelhout et al. [55] but adapted for the purposes of this review.

### Synthesis

We aim to refine our program theory by identifying which outcomes are caused by the mechanisms, which specific mechanisms serve to explain these effects, and which contextual factors play a role in whether the mechanisms are activated. We will therefore seek data from the included studies to test these elements of our program theory and investigate whether there are recurring patterns. In order to synthesize the data, we will use the output of the assessment on relevance and rigor. The first author (ML) will be in charge of this main part of the analysis. Findings will be presented to and discussed with the second (FJL) and last author (GEN). During this process, the focus will be on the interpretation of meaning of the data. If sections of documents are considered both relevant and trustworthy, we will evaluate whether the data can be interpreted as functioning as context, mechanism, or outcome. In addition, we will evaluate whether the data provide evidence for any (partial or complete) context-mechanism-outcome configurations and whether the data justify changing or corroborating (elements of) the program theory. In order to do so, we will not only look at these relationships within each document, but also across documents (using the coded data in NVivo). If necessary, we will iteratively search for additional data to test (elements of) the refined program theory. This could for instance entail documents of studies in other areas of mental health care in which certain relationships between contextual factors, mechanisms, and outcomes have been established. We will make use of several approaches to synthesizing the data, which include juxtaposing, consolidating, reconciling, adjudicating, and situating sources of evidence [47]. The findings might explain or complement one another, which will make it possible to build a multi-faceted explanation of success. They might, however, also contradict each other, despite similar circumstances, which will necessitate seeking an explanation. Judging studies on the basis of their methodological quality might allow for a preference for one explanation over another. Lastly, comparing studies in comparative settings will provide information on which contextual factors are important.

Finally, we will judge the coherence of the theory by looking at three aspects: consilience, simplicity, and analogy. Coherence therefore refers to whether the theory is able to explain as much as possible of the data, whether the theory is simple and does not need additional assumptions to be able to explain the data, and whether the theory fits with our current knowledge or substantive theory [53].

The results of the synthesis will be discussed with the review team and other experts to assess the validity and relevance. We will be careful to take into account the overall body of evidence and pay attention to the quality and the balance between desirable and undesirable effects. Based on the findings, the program theory will be refined into a final model. The results of the analysis and synthesis will be described in accordance with the standard for reporting realist reviews, RAMESES [49]. RAMESES includes guidelines for describing the rationale for the review, any changes that were made to the review process, (the rationale regarding) the iterative search, how judgments were made regarding the selection and appraisal of papers, and the key findings.

## Discussion

A realist approach will be utilized in the study described in this protocol in order to investigate the effects of support by experiential peers in relation to desistance and desistance-related outcomes. This type of systematic review allows for exploring mechanisms through which these effects occur and contextual factors that might influence these processes, thereby providing a more complete and informative account of these types of interventions aimed at individuals involved in delinquent behavior. In this protocol paper, we presented our initial program theory, which includes seven mechanisms: (1) empathy and acceptance, (2) social learning, (3) social bonding, (4) social control, (5) narrative and identity formation, (6) hope and perspective, and (7) translation and connection.

The realist review approach ideally provides evidence for specific context-mechanism-outcome (CMO) configurations. However, it is plausible that many researchers examine a combination of mechanisms or outcomes, making the uncovering of separate CMO configurations impossible. In addition, the review will not lead to a conclusive answer regarding what makes support by experiential peers potentially effective; other theoretical explanations of how interventions with experiential expertise lead to certain outcomes could be postulated. By involving a multidisciplinary team (with backgrounds in pedagogy, criminology, psychology, sociology, and public health) and the perspectives of experiential peers in the development of the initial model, we try to consider a wide range of viewpoints. Another challenge with realist reviews is that the iterative approach used can go on indefinitely without a predefined endpoint. In order to minimize this risk, we will thoroughly prepare the second literature search by discussing it with our team and consulting experts.

The findings of the realist review will contribute to the current knowledge on effects and mechanisms of support by experiential peers in general, and in the forensic setting in particular. By offering an overview of evidence-based mechanisms involved in such interventions, we will provide insight into which aspects remain to be studied. The findings might help professional care providers to know whether (more) effort should be put into involving experiential peers in reaching individuals involved in delinquent behavior and supporting them in their desistance process. It might also provide them with information on the conditions under which these interventions especially lead to positive results. In addition, this study might provide professional care providers lacking such experiential similarity to the clients they are serving with tools to improve their relationship with them by learning from experiential peers. Lastly, it might provide policymakers guidance in the allocation of funding to projects making use of experiential expertise. By disseminating the findings of the realist review to policymakers and other stakeholders, we aim to contribute to the implementation of evidence-based interventions to improve outcomes for individuals who engage in delinquent behavior and to support them in the process of desistance.

## Additional files

Additional file 1: PRISMA-P 2015 Checklist. (DOCX 31 kb)

Additional file 2: Search strategy. (DOCX 20 kb)

## Abbreviations

CMO: Context-mechanism-outcome
DUI: Driving under the influence
PRISMA-P: Preferred Reporting Items for Systematic Review and Meta-Analysis Protocols
RAMESES: Realist and Meta-Review Evidence Synthesis: Evolving Standards
