# Supplementary material for: Experiential peer support and its effects on desistance from delinquent behavior: protocol paper for a systematic realist literature review
Source: Syst Rev. 2019 May 18;8:119. doi: 10.1186/s13643-019-1036-2 (PMC6525969; doi:10.1186/s13643-019-1036-2)
Supplement: Supplementary file 2 — Search strategy. (DOCX 20 kb) [file 13643_2019_1036_MOESM2_ESM.docx]

# Additional file 2

## Search strategy

***Embase.com (Embase incl. Medline): 1008***

('offender'/de OR 'crime'/exp OR 'conduct disorder'/de OR 'antisocial personality disorder'/de OR *'delinquency'/exp OR 'recidivism'/de OR 'prisoner'/exp* OR (*((conduct OR 'antisocial personality') NEXT/3 (disorder*))* OR delinquen* OR recidivis* OR criminal* OR crime* *OR* offender* OR convict* OR perpetrator* *OR prisoner* OR desistance* OR felon* OR detainee* OR inmate* OR incarcerated OR* *parole* OR justice-involved*):ab,ti) **AND** **(**'caregiver'/de OR 'community care'/exp OR 'social care'/de OR 'psychosocial care'/de OR 'social work'/de *OR 'behavioral health care'/de OR 'mental health service'/de OR 'peer counseling'/de* *OR 'counseling'/de* *OR ‘aftercare’/de* OR 'probation'/de *OR 'prison'/de OR 'criminal justice'/de OR 'juvenile court'/de OR 'reintegration'/de OR 'correctional institution'/de OR 'incarceration'/de OR 'imprisonment'/de* OR 'community reintegration'/de OR 'community based rehabilitation'/de OR 'experiential education'/de *OR 'vocational rehabilitation'/de OR 'rehabilitation care'/de OR 'support group'/de OR 'group counseling'/de OR 'caregiving'/de* OR (caregiv* OR ((care) NEAR/3 (giver* OR giving* OR provider* OR community OR network*)) OR ((behaviour* *OR behavior* OR mental*) *NEXT/3* (care OR service*)) OR ((social *OR psychosocial** *OR psychiatr* OR psycholog**) NEXT/1 (service* OR case OR work* OR care)) *OR carer** OR probation* OR *((community) NEXT/3 (network** *OR project OR service**)) OR ((*self-help* OR selfhelp OR *support*) *NEXT/1* (group*)) OR ((peer) NEAR/3 (mentor* OR interven* OR support* *OR approach* OR counsel* OR program**)) OR rehabilitat* OR recover* OR reintegrat* *OR prison* OR penitentiar* OR jail* OR forensic OR desistance OR incarcerat* OR detention OR parole* OR aftercare OR ((youth OR outreach) NEXT/1 (work)) OR ((criminal OR juvenile) NEXT/1 (justice OR court)) OR imprison* OR ((correctional OR penal) NEXT/1 (institution* OR facilit*)) OR ((group) NEXT/1 (intervention*))* OR ((experiential) NEXT/1 (learning OR education)) OR ((diversion *OR recovery OR re-entry OR reentry OR outreach*) NEAR/3 (program*))):ab,ti**)** **AND** **(**'peer counseling'/de OR 'experiential education'/de *OR ‘support group’/de OR ‘personal experience’/de* OR (((peer*) NEAR/3 (support* OR help* *OR counsel** OR led OR based OR deliver* OR educat* OR worker* OR mentor* OR interven* OR tutor* OR mediat* OR advoca* OR advisor* OR navigator* OR provide* *OR specialist* OR teach* OR facilitat* OR practice* OR model* OR approach**)) OR ((experien*) *NEAR/3* (expert* OR lived OR knowledge OR education *OR learning*)) OR ((mutual) *NEXT/1* (support* OR help* *OR aid**)) OR ((*self-help* OR selfhelp *OR support*) NEXT/1 (group*))):ab,ti**) NOT (**'Conference Abstract' OR Editorial**)**/it

***Medline Epub (Ovid): 1107***

(*Antisocial Personality Disorder/* OR Criminals/ OR exp Crime/ *OR Juvenile Delinquency/* OR Conduct *Disorder/ OR Prisoners/* OR (*((conduct OR "antisocial personality") ADJ3 (disorder*))* OR delinquen* OR recidivis* OR criminal* OR crime* *OR* offender* OR convict* OR perpetrator* *OR prisoner* OR desistance* OR felon* OR detainee* OR inmate* OR incarcerated OR parole* OR justice-involved*).ab,ti.) **AND** **(**Caregivers/ OR Community Networks/ OR exp Social Work/ *OR Self-Help Groups/ OR exp Mental Health Services/* OR Community Reintegration/ OR Rehabilitation/ *OR Psychiatric Rehabilitation/* OR Experiential Education/ *OR Prisons/ OR Counseling/ OR Rehabilitation, Vocational/* OR (caregiv* OR ((care) ADJ3 (giver* OR giving* OR provider* OR community OR network*)) OR ((behaviour* OR behavior* OR mental) *ADJ3* (care OR service*)) OR ((social OR psychosocial* *OR psychiatr* OR psycholog**) *ADJ1* (service* OR case OR work* OR care)) *OR carer** OR probation* OR *((community) ADJ3 (network** *OR project OR service**)) OR ((self-help OR selfhelp OR *support*) ADJ1 (group*)) OR ((peer) ADJ3 (mentor* OR interven* OR support* OR approach* OR counsel* OR program*)) OR rehabilitat* OR recover* OR reintegrat* *OR prison* OR penitentiar* OR jail* OR forensic OR desistance OR incarcerat* OR detention OR parole* OR aftercare OR ((youth OR outreach) ADJ1 (work)) OR ((criminal OR juvenile) ADJ1 (justice OR court)) OR imprison* OR ((correctional OR penal) ADJ1 (institution* OR facilit*))* OR ((experiential) ADJ1 (learning OR education)) OR ((diversion *OR recovery OR re-entry OR reentry OR outreach*) ADJ3 (program*))).ab,ti.**)** **AND** **(***Self-Help Groups/* OR (((peer*) ADJ3 (support* OR help* *OR counsel** OR led OR based OR deliver* OR educat* OR worker* OR mentor* OR interven* OR tutor* OR mediat* OR advoca* OR advisor* OR navigator* OR provide* *OR specialist* OR teach* OR facilitat* OR practice* OR model* OR approach**)) OR ((experien*) *ADJ3* (expert* OR lived OR knowledge OR education *OR learning*)) OR ((mutual) ADJ1 (support* OR help* *OR aid**)) OR ((self-help OR selfhelp *OR support*) ADJ1 (group*))).ab,ti.**) NOT (**congresses OR editorial**)**.pt.

***PsycInfo (Ovid): 1831***

(*Antisocial Personality Disorder/ OR exp Perpetrators/* OR exp Crime/ *OR exp Criminal Behavior/* OR Conduct *Disorder/ OR Prisoners/* OR Recidivism/ OR Parolees/ OR (*((conduct OR "antisocial personality") ADJ3 (disorder*))* OR delinquen* OR recidivis* OR criminal* OR crime* *OR* offender* OR convict* OR perpetrator* *OR prisoner* OR desistance* OR felon* OR detainee* OR inmate* OR incarcerated OR parole* OR justice involved*).ab,ti.) **AND** **(**Caregivers/ OR Community Networks/ OR exp Social Services/ *OR Support Groups/ OR exp Mental Health Services/* *OR Counseling/ OR Group Counseling/ OR Peer Counseling/ OR Rehabilitation Counseling/* OR Reintegration/ OR Rehabilitation/ OR Parole/ *OR exp Psychosocial Rehabilitation/ OR Criminal Rehabilitation/ OR exp Correctional Institutions/ OR Outreach Programs/* OR (caregiv* OR ((care) ADJ3 (giver* OR giving* OR provider* OR community OR network*)) OR ((behaviour* OR behavior* OR mental) *ADJ3* (care OR service*)) OR ((social OR psychosocial* *OR psychiatr* OR psycholog**) *ADJ1* (service* OR case OR work* OR care)) *OR carer** OR probation* OR *((community) ADJ3 (network** *OR project OR service**)) OR ((self help OR selfhelp OR *support*) ADJ1 (group*)) OR ((peer) ADJ3 (mentor* OR interven* OR counsel* OR support* OR approach* OR counsel* OR program*)) OR rehabilitat* OR recover* OR reintegrat* *OR prison* OR penitentiar* OR jail* OR forensic OR desistance OR incarcerat* OR detention OR parole* OR aftercare OR ((youth OR outreach) ADJ1 (work)) OR ((criminal OR juvenile) ADJ1 (justice OR court)) OR imprison* OR ((correctional OR penal) ADJ1 (institution* OR facilit*)) OR ((group) ADJ1 (intervention OR counseling))* OR ((experiential) ADJ1 (learning OR education)) OR ((diversion *OR recovery OR re-entry OR reentry OR outreach*) ADJ3 (program*))).ab,ti.**)** **AND** **(***Peer Tutoring/ OR Peer Counseling/* OR (((peer*) ADJ3 (support* OR help* OR counsel* OR led OR based OR deliver* OR educat* OR worker* OR mentor* OR interven* OR tutor* OR mediat* OR advoca* OR advisor* OR navigator* OR provide* *OR specialist* OR teach* OR facilitat* OR practice* OR model**)) OR ((experien*) *ADJ3* (expert* OR lived OR knowledge OR education *OR learning*)) OR ((mutual) ADJ1 (support* OR help* *OR aid**)) OR ((self-help OR selfhelp *OR support*) ADJ1 (group*))).ab,ti.**) NOT (**congresses OR editorial**)**.pt.

***Cochrane Central (trials): 49***

((*((conduct OR "antisocial personality") NEXT/3 (disorder*))* OR delinquen* OR recidivis* OR criminal* OR crime* *OR* offender* OR convict* OR perpetrator* *OR prisoner* OR desistance* OR felon* OR detainee* OR inmate* OR incarcerated OR parole* OR justice-involved*):ab,ti) **AND** **(**(caregiv* OR ((care) NEAR/3 (giver* OR giving* OR provider* OR community OR network*)) OR ((behaviour* *OR behavior* OR mental*) *NEXT/3* (care OR service*)) OR ((social *OR psychosocial** *OR psychiatr* OR psycholog**) NEXT/1 (service* OR case OR work* OR care)) *OR carer** OR probation* OR *((community) NEXT/3 (network** *OR project OR service**)) OR (("self help" OR selfhelp OR *support*) NEXT/1 (group*)) OR ((peer) NEAR/3 (mentor* OR interven* OR support* *OR approach* OR counsel* OR program**))OR rehabilitat* OR recover* OR reintegrat* *OR prison* OR penitentiar* OR jail* OR forensic OR desistance OR incarcerat* OR detention OR parole* OR aftercare OR ((youth OR outreach) NEXT/1 (work)) OR ((criminal OR juvenile) NEXT/1 (justice OR court)) OR imprison* OR ((correctional OR penal) NEXT/1 (institution* OR facilit*)) OR ((group) NEXT/1 (intervention*))* OR ((experiential) NEXT/1 (learning OR education)) OR ((diversion *OR recovery OR re-entry OR reentry OR outreach*) NEAR/3 (program*))):ab,ti**)** **AND** **(**(((peer*) NEAR/3 (support* OR help* OR counsel* OR led OR based OR deliver* OR educat* OR worker* OR mentor* OR interven* OR tutor* OR mediat* OR advoca* OR advisor* OR navigator* OR provide* *OR specialist* OR teach* OR facilitat* OR practice* OR model* OR approach**)) OR ((experien*) NEAR/3 (expert* OR lived OR knowledge OR education *OR learning*)) OR ((mutual) NEXT/1 (support* OR help* *OR aid**)) OR ((self-help OR selfhelp *OR support*) NEXT/1 (group*))):ab,ti**)**

***Web of Science (Core Collection all sciences): 1012***

**TS=(**((*((conduct OR "antisocial personality") NEAR/2 (disorder*))* OR delinquen* OR recidivis* OR criminal* OR crime* *OR* offender* OR convict* OR perpetrator* *OR prisoner* OR desistance* OR felon* OR detainee* OR inmate* OR incarcerated OR parole* OR justice-involved*)) **AND** **(**(caregiv* OR ((care) NEAR/2 (giver* OR giving* OR provider* OR community OR network*)) OR ((behaviour* *OR behavior* OR mental*) *NEAR/2* (care OR service*)) OR ((social *OR psychosocial** *OR psychiatr* OR psycholog**) *NEAR/1* (service* OR case OR work* OR care)) *OR carer** OR probation* OR *((community) NEAR/2 (network** *OR project OR service**)) OR ((self-help OR selfhelp OR *support*) NEAR/1 (group*)) OR ((peer) NEAR/2 (mentor* OR interven* OR support* *OR approach* OR counsel* OR program**)) OR rehabilitat* OR recover* OR reintegrat* *OR prison* OR penitentiar* OR jail* OR forensic OR desistance OR incarcerat* OR detention OR parole* OR aftercare OR ((youth OR outreach) NEAR/1 (work)) OR ((criminal OR juvenile) NEAR/1 (justice OR court)) OR imprison* OR ((correctional OR penal) NEAR/1 (institution* OR facilit*)) OR ((group) NEAR/1 (intervention*))* OR ((experiential) NEAR/1 (learning OR education)) OR ((diversion *OR recovery OR re-entry OR reentry OR outreach*) NEAR/2 (program*)))) AND ((((peer*) NEAR/2 (support* OR help* OR counsel* OR led OR based OR deliver* OR educat* OR worker* OR mentor* OR interven* OR tutor* OR mediat* OR advoca* OR advisor* OR navigator* OR provide* *OR specialist* OR teach* OR facilitat* OR practice* OR model* OR approach**)) OR ((experien*) NEAR/2 (expert* OR lived OR knowledge OR education *OR learning*)) OR ((mutual) NEAR/1 (support* OR help* *OR aid**)) OR ((self-help OR selfhelp *OR support*) NEAR/1 (group*)))**))** ***AND*** DT=Article

***Scopus: 1652***

**TITLE-ABS-KEY(**((*((conduct OR "antisocial personality") PRE/2 (disorder*))* OR delinquen* OR recidivis* OR criminal* OR crime* *OR* offender* OR convict* OR perpetrator* *OR prisoner* OR desistance* OR felon* OR detainee* OR inmate* OR incarcerated OR parole* OR "justice-involved"*)) **AND** **(**(caregiv* OR ((care) W/2 (giver* OR giving* OR provider* OR community OR network*)) OR ((behaviour* *OR behavior* OR mental*) *PRE/2* (care OR service*)) OR ((social *OR psychosocial** *OR psychiatr* OR psycholog**) *PRE/1* (service* OR case OR work* OR care)) *OR carer** OR probation* OR *((community) PRE/2 (network** *OR project OR service**)) OR ((self-help OR selfhelp OR *support*) *PRE/1* (group*)) OR ((peer) W/2 (mentor* OR interven* OR support* *OR approach* OR counsel* OR program**)) OR rehabilitat* OR recover* OR reintegrat* *OR prison* OR penitentiar* OR jail* OR forensic OR desistance OR incarcerat* OR detention OR parole* OR aftercare OR ((youth OR outreach) PRE/1 (work)) OR ((criminal OR juvenile) PRE/1 (justice OR court)) OR imprison* OR ((correctional* OR penal*) PRE/1 (institution* OR facilit*)) OR ((group) PRE/1 (intervention*))* OR ((experiential) PRE/1 (learning OR education)) OR ((diversion *OR recovery OR re-entry OR reentry OR outreach*) W/2 (program*)))**)** **AND** **(**(((peer*) PRE/2 (support* OR help* OR counsel* OR led OR based OR deliver* OR educat* OR worker* OR mentor* OR interven* OR tutor* OR mediat* OR advoca* OR advisor* OR navigator* OR provide* *OR specialist* OR teach* OR facilitat* OR practice* OR model* OR approach**)) OR ((experien*) W/2 (expert* OR lived OR knowledge OR education *OR learning*)) OR ((mutual) PRE/1 (support* OR help* *OR aid**)) OR ((self-help OR selfhelp *OR support*) PRE/1 (group*)))**))**

***Limit to document Type:*** *Article OR Review*

***Criminal Justice Abstracts: 352***

*Fieldnames searched: AB OR TI OR SU OR KW*

("offender" OR "crime" OR "conduct disorder" OR "antisocial personality disorder" OR "delinquen*" OR "recidivis*" OR "criminal*" OR "convict*" OR "perpetrator*" OR "prisoner*" OR "desistance" OR "felon*" OR "detainee*" OR "inmate*" OR "incarcerated" OR "parolee*" OR "justice-involved")

**AND**

("caregiv*" OR "care provider" OR "community care" OR "community project" OR "community network*" OR "community service*" OR "mental health service*" OR "mental health care" OR "behavioral health care" OR "behavioral health service*" OR "social care" OR "psychosocial care" OR "psychiatr* care" OR "psycholog* care" OR "social service*" OR "social case work" OR "social work*" OR "care network" OR "probation" OR "prison*" OR "penitentiar*" OR "jail" OR "forensic" OR "criminal justice" OR "recovery program*" OR "desistance" OR "juvenile justice" OR "juvenile court" OR "correctional institution" OR "correction facilit*" OR "penal institution" OR "penal facility*" OR "incarcerat*" OR "detention*" OR "imprison*" OR "parole" OR "aftercare" OR "youth work*" OR "outreach work*" OR "experiential education" OR "experiential learning" OR "care provider*" OR "carer*" OR "peer counsel*" OR "peer mentor*" OR "peer interven*" OR "self help group" OR "selfhelp group" OR "rehabilitat*" OR "reintegrat*" OR "recover*" OR "diversion program*" OR "recovery program*" OR "re-entry program*" OR "reentry program*" OR "outreach program*" OR "support group*" OR "group counsel*" OR "group intervention" OR "peer support*" OR "peer approach*" OR “peer program*”)

**AND**

("peer teach*" OR "experiential learning" OR "support group*" OR "experiential education" OR "peer support*" OR "peer help*" OR "peer counsel*" OR "peer led" OR "peer educat*" OR "peer worker" OR "peer mentor*" OR "peer interven*" OR "peer tutor" OR "peer mediat*" OR "peer based" OR "peer deliver*" OR "peer advoca*" OR "peer advisor*" OR "peer navigator*" OR "peer provide*" OR "peer specialist" OR "peer facilitat*" OR "peer practice*" OR "peer model*" OR “peer role model*" OR "peer approach*" OR "experien* expert" OR "expert experience" OR "experiential knowledge" OR "lived experience" OR "mutual support" OR "mutual help" OR "mutual aid" OR "self help group" OR "selfhelp group")

***SOCIndex: 765***

[SU] (((((((((((((DE "DELINQUENT behavior") OR (DE "CRIME")) OR (DE "CONDUCT disorders in adolescence")) OR (DE "ANTISOCIAL personality disorders")) OR (DE "RECIDIVISM")) OR (DE "RECIDIVISTS")) OR (DE "CRIMINALS")) OR (DE "EX-convicts")) OR (DE "PRISONERS")) OR (DE "PAROLEES")) OR (DE "DESISTANCE from crime")) OR (DE "CRIMINAL behavior")) OR (DE "JUVENILE delinquency")) ***OR*** [AB OR TI OR KW] ("offender" OR "crime" OR "conduct disorder" OR "antisocial personality disorder" OR "delinquen*" OR "recidivis*" OR "criminal*" OR "convict*" OR "perpetrator*" OR "prisoner*" OR "desistance" OR "felon*" OR "detainee*" OR "inmate*" OR "incarcerated" OR "parolee*" OR "justice-involved*")

**AND**

[SU] ((((((((((((((((((((((((DE "CAREGIVERS") OR (DE "SOCIAL case work")) OR (DE "SOCIAL support")) OR (DE "SOCIAL services")) OR (DE "COMMUNITY mental health services")) OR (DE "MENTAL health services")) OR (DE "PROBATION")) OR (DE "COUNSELING")) OR (DE "REHABILITATION")) OR (DE "JUVENILE delinquents -- Rehabilitation")) OR (DE "REHABILITATION of criminals")) OR (DE "JUVENILE courts")) OR (DE "CORRECTIONAL institutions")) OR (DE "JUVENILE detention")) OR (DE "DETENTION of persons")) OR (DE "imprisonment")) OR (DE "DESISTANCE from crime")) OR (DE "VOCATIONAL rehabilitation")) OR (DE "jails")) OR (DE "DIVERSION programs")) OR (DE "PAROLE")) OR (DE "AFTERCARE services")) OR (DE "EXPERIENTIAL learning")) OR (DE "SUPPORT groups")) OR (DE "GROUP counseling")) ***OR*** [AB OR TI OR KW] ("caregiv*" OR "care provider" OR "community care" OR "community project" OR "community network*" OR "community service*" OR "mental health service*" OR "mental health care" OR "behavioral health care" OR "behavioral health service*" OR "social care" OR "psychosocial care" OR "psychiatr* care" OR "psycholog* care" OR "social service*" OR "social case work" OR "social work*" OR "care network" OR "probation" OR "prison*" OR "jail" OR "penitentiar*" OR "forensic" OR "criminal justice" OR "recovery program*" OR "desistance" OR "juvenile justice" OR "juvenile court" OR "correctional institution" OR "correction facilit*" OR "penal institution" OR "penal facilit*" OR "incarcerat*" OR "detention*" OR "imprison*" OR "parole" OR "aftercare" OR "youth work*" OR "outreach work*" OR "experiential education" OR "experiential learning" OR "care provider*" OR "carer*" OR "peer counsel*" OR "peer mentor*" OR "peer interven*" OR "self help group" OR "selfhelp group" OR "rehabilitat*" OR "reintegrat*" OR "recover*" OR "diversion program*" OR "recovery program*" OR "re-entry program*" OR "reentry program*" OR "outreach program*" OR "support group*" OR "group counseling" OR "group intervention" OR "peer support*" OR "peer approach*" OR “peer program*”)

**AND**

[SU] ((((DE "PEER teaching") OR (DE "EXPERIENTIAL learning")) OR (DE "MUTUAL aid")) OR (DE "SUPPORT groups")) ***OR*** [AB OR TI OR KW] ("peer teach*" OR "experiential learning" OR "support group*" OR "experiential education" OR "peer support*" OR "peer help*" OR "peer counsel*" OR "peer led" OR "peer educat*" OR "peer worker" OR "peer mentor*" OR "peer interven*" OR "peer tutor" OR "peer mediat*" OR "peer based" OR "peer deliver*" OR "peer advoca*" OR "peer advisor*" OR "peer navigator*" OR "peer provide*" OR "peer specialist" OR "peer facilitat*" OR "peer practice*" OR "peer model*" OR “peer role model*" OR "experien* expert" OR "expert experience" OR "peer approach" OR "experiential knowledge" OR "lived experience" OR "mutual support" OR "mutual help" OR "mutual aid" OR "self help group" OR "selfhelp group")

***Google Scholar: 200 (****top relevant refs)*

delinquent|delinquency|criminal|crime|offender caregiver|*probation|rehabilitation*|"social|psychosocial care"|"peer|group counseling|mentor|*intervention|support***"** "peer counseling|*intervention|support|mentor"*
